# Rare presentation of Graves’ disease with myalgia: A case report

**DOI:** 10.1002/ccr3.4629

**Published:** 2021-10-08

**Authors:** Masaru Kurihara, Shunichi Kinjo, Yasuharu Tokuda

**Affiliations:** ^1^ Department of Hospital Medicine Urasoe General Hospital Okinawa Japan; ^2^ Muribushi Okinawa Center for Teaching Hospitals Okinawa Japan

**Keywords:** endocrine disease, Graves’ disease, myalgia

## Abstract

A 42‐year‐old woman presented with myalgia, which ameliorated a week after treatment. She was diagnosed with Graves’ disease. The presence of concomitant autoimmune diseases is important considerations for patients with Graves’ disease presenting with myalgia. Thyrotoxicosis should be included as a rare differential diagnosis for myalgia.

## Graphical abstract

It is important to consider the disease mechanism and the presence of other concomitant diseases with Graves’ disease. Thyrotoxicosis could be a differential diagnosis in patients who present with myalgia.

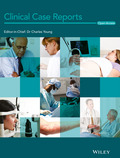

## 1 INTRODUCTION

Graves’ disease is the most common cause of thyrotoxicosis.^1^ Thyrotropin receptor antibody (TRAb) is produced on the surface of the thyroid gland as an autoantibody against the binding receptor of thyroid‐stimulating hormone (TSH). TRAb overstimulates the TSH receptor instead of TSH, causing excessive production of thyroid hormones that subsequently causes Graves’ disease.^1^ Abnormally high levels of thyroid hormones that increase the metabolism of the entire body can have significant health consequences. Known autoimmune mechanisms include orbital disease, goiter, and thyroid dermopathy.^2^ Symptoms of Graves’ disease can be caused by either hyperthyroidism or autoimmune mechanisms. Hypothyroidism commonly causes myalgia in thyroid gland dysfunction.^3^ Herein, we report a case of Graves’ disease that presented with a rare complaint of myalgia.

## 2 CASE HISTORY

A 42‐year‐old Japanese woman presented with a 4‐week history of myalgia. Two weeks before her visit to our hospital, her pain began to spread from the neck to the chest, resulting in extreme myalgia four days prior to her visit. The pain was sudden, intermittent, and generalized with no clear localization. There was no change in her symptoms owing to diet. The patient reported excessive sweating and weight loss (5 kg/6 months); however, tremors, diarrhea, and palpitation were absent. Though her medical history included pyelonephritis, she had no remarkable family history. Furthermore, she was not taking any oral medications or supplements.

On examination, the patient appeared ill and was afebrile (37.0℃). Her blood pressure was 110/64 mmHg, pulse 90/min, respiratory rate 16/min with an O_2_ saturation of 98% on room air. Physical examination revealed no ocular protrusion or eye movement disorder. Her thyroid gland was diffusely enlarged, but there was no associated tenderness or nodule present on physical examination. Her cardiac examination results were normal, and lungs were clear on auscultation. There were no abnormal abdominal findings. Findings of neurological examinations, including tests for muscle weakness, muscle grasping pain, muscle atrophy, and deep tendon reflexes, were completely unremarkable.

On admission, initial investigations revealed mild anemia. Inflammatory markers and kidney function test results were normal, but liver function test values were mildly elevated. Creatinine kinase, serum calcium, and serum phosphate levels were normal. Further examination revealed free triiodothyronine (FT3) >30.00 ng/ml, free thyroxine (FT4) 3.45 ng/ml, TSH <0.01 µIU/ml, and findings of thyrotoxicosis. Additionally, she tested positive for TRAb (6.4 IU/L). The results of tests for autoantibodies, including antinuclear antibodies, were negative (Table 1).

**TABLE 1:** Laboratory data

Variable	Reference range	On admission
Hemoglobin (g/dl)	11.6–14.8	10.8
Hematocrit (%)	35.1–44.4	33.5
White blood cell count (per μl)	3300–8600	3500
Differential count (per μl)		
Neutrophils (%)	0.0–2.5	39.0
Lymphocytes (%)	16.5–49.5	43.8
Monocytes (%)	2.0–10.0	14.1
Eosinophil (%)	0.0–8.5	2.8
Platelet count (per μl)	158,000–348,000	221,000
Sodium (mmol/L)	135–145	141
Potassium (mmol/L)	3.6–4.8	4.2
Chloride (mmol/L)	101–108	109
Calcium (mg/dl)	8.8–10.1	9.1
Inorganic phosphate (mg/dl)	2.4–4.3	2.7
Urea nitrogen (mg/dl)	8.0–20.0	14.7
Creatinine (mg/dl)	0.46–0.79	0.49
Alanine aminotransferase (U/L)	13–30	50
Aspartate aminotransferase (U/L)	7–23	93
Alkaline phosphatase (U/L)	106–322	217
Creatine kinase (U/L)	41–153	138
Lactate dehydrogenase (U/L)	124–222	194
C‐reactive protein (mg/dl)	0.0–0.1	0.0
FT3 (ng/ml)	1.71–3.71	>30.0
FT4 (ng/ml)	0.70–1.48	3.45
TSH (µIU/ml)	0.35–4.94	<0.01
TRAb (IU/L)	<2.0	6.4
Anti‐thyroglobulin antibody	<28.0	383.0
Anti‐thyroid peroxidase antibodies	0.0–15.9	14.3
TSAb (%)	<180	280
Thyroglobulin (ng/ml)	<45	90.0
Antinuclear antibody	<40	Negative
Anti‐aminoacyl tRNA synthetase antibody	<25	Negative
Rheumatoid factor (U/ml)	0.0–15.0	0.0

Abbreviations: FT3, free triiodothyronine; FT4, free thyroxine; RNA, ribonucleic acid; TRAb, thyrotropin receptor antibody; TSAb, thyroid‐stimulating antibody; TSH, thyroid‐stimulating hormone (thyrotropin).

Cervical ultrasonography showed diffuse enlargement of the thyroid gland and increased blood flow with a color Doppler scan of 57.6 cm/s and 48.1 cm/s on the right and left, respectively. Consequently, she was diagnosed with Graves’ disease. Since there were no specific symptoms and autoantibodies were absent, we concluded that there was no complication of myositis. Treatment with mercazole 15 mg and potassium iodide 50 mg was initiated, leading to an improvement in FT3 (>4.49 ng/ml) and FT4 (1.31 ng/ml) levels after one month of treatment. Myalgia disappeared one week after the administration of the anti‐thyroid medication. Potassium iodide was discontinued once FT4 was normalized. Thereafter, the condition of the patient improved without any relapse of pain after the anti‐thyroid medication withdrawal.

## 3 DISCUSSION

Our patient had Graves’ disease and presented with the rare chief complaint of myalgia. Musculoskeletal symptoms and signs are common in patients with thyroid dysfunction, as the skeletal muscle is a major target of thyroid hormones.^4^ Although hypothyroidism is a thyroid function abnormality associated with myalgia, hyperthyroidism is mainly associated with myopathy, such as muscle weakness and wasting. Symptoms of myopathy primarily involve the proximal muscles and rarely the pectoralis major muscle.^5^ Hyperthyroid myopathy usually resolves after recovery from hyperthyroidism. In the present case, the findings were atypical because there was no muscle weakness, no muscular symptoms, and myalgia was not localized to the muscles. Since myalgia in cases such as the presented one is very rare, careful consideration of the complications of concomitant disease is needed, followed by a detailed medical interview, review of the system, and systemic physical examination.

In recent years, cases of myalgia and elevated creatine kinase levels during the treatment of hyperthyroidism have been presented, and the side effects of anti‐thyroid drugs and relative hypothyroidism have been proposed as explanations for these muscle symptoms.^5^ However, the presented case is not consistent with relative hypothyroidism because myalgia was present before treatment. Because T4 abnormalities cause abnormal glycogen degradation, abnormal mitochondrial oxidative metabolism, and abnormal triglyceride turnover, which impair muscle function,^6^ it is usually seen in hypothyroidism, but may be occurred in hyperthyroidism as well. Muscle symptoms caused by Graves's disease require attention as they could be complications of other autoimmune diseases. Graves’ disease is rarely associated with other autoimmune diseases, such as polymyositis and myasthenia gravis.^7^, ^8^ Further, the absence of other features of muscle fatigue and myasthenia gravis in the present case ruled out other autoimmune diseases. Similarly, periodic limb paralysis is a rare and life‐threatening complication of Graves’ disease.^9^ It is characterized by recurrent episodes of transient, flaccid muscle paralysis and affects the proximal muscles more severely than distal muscles. The clinical course of the presented case and normal serum potassium levels were inconsistent with periodic quadriplegia. Furthermore, vitamin D deficiency can be an etiological factor for Graves’ disease and non‐specific musculoskeletal pain.^10^, ^11^ However, this possibility was ruled out because there was no evidence of vitamin D deficiency in the present case. As in previous reports,^12^ the improvement in myalgia as well as the simultaneous recovery of hyperthyroidism strongly suggests that hyperthyroidism was the cause of the myalgia. To the best of our knowledge, this is a very rare symptom of Graves’ disease and has only been reported in two previous cases.^9^

In conclusion, we report on a patient with Graves’ disease who presented with the chief complaint of myalgia, which is a rare symptom. In patients with Graves’ disease who complain of muscular symptoms, it is important to consider the disease mechanism as well as the presence of other concomitant diseases; thyrotoxicosis could be a differential diagnosis in such patients who present with myalgia.

## CONFLICT OF INTEREST

None. We had no Conflict of Interest.

## AUTHOR CONTRIBUTIONS

All authors critically revised the report, commented on drafts of the manuscript, and approved the final report.

## ETHICAL APPROVAL

We have obtained informed consent from the patient for publication of this case report.

## INFORMED CONSENT

We obtained informed consent from the patient for publication of this case report.

## Data Availability

The datasets used and/or analyzed during the current study are available from the corresponding author on reasonable request.
